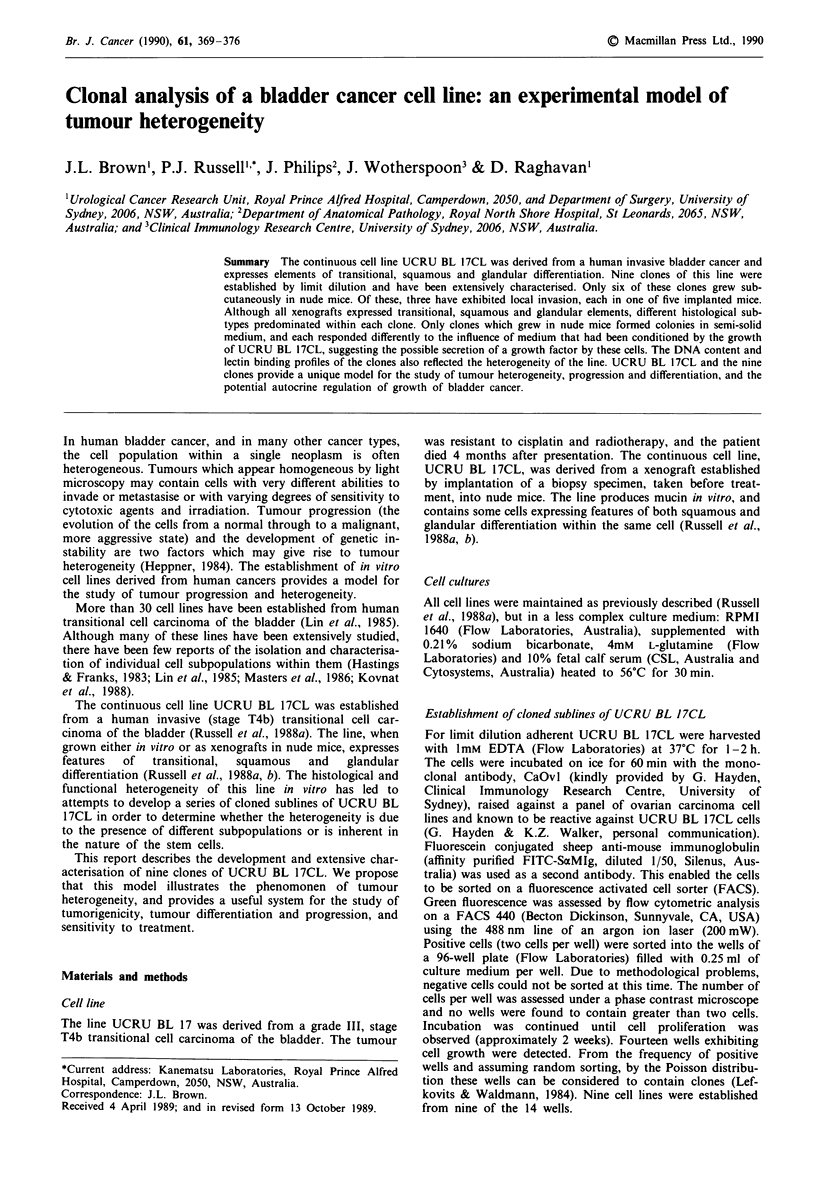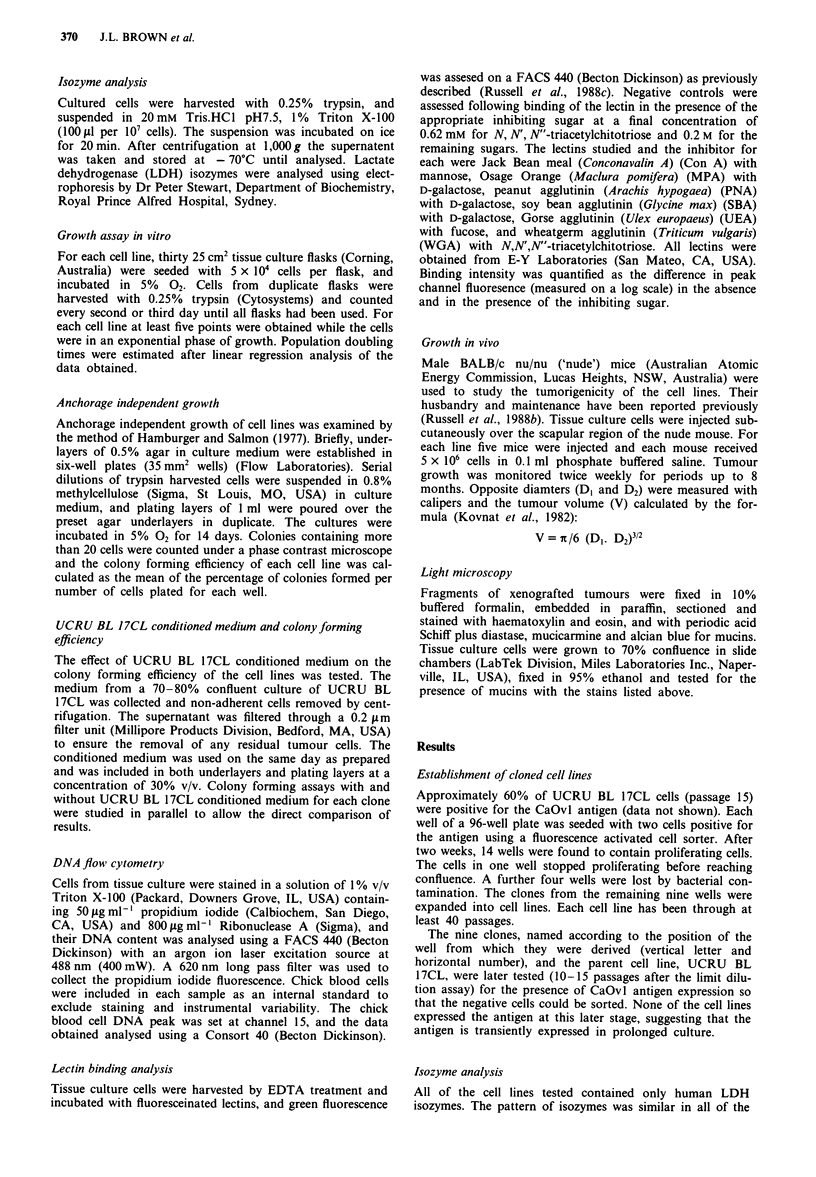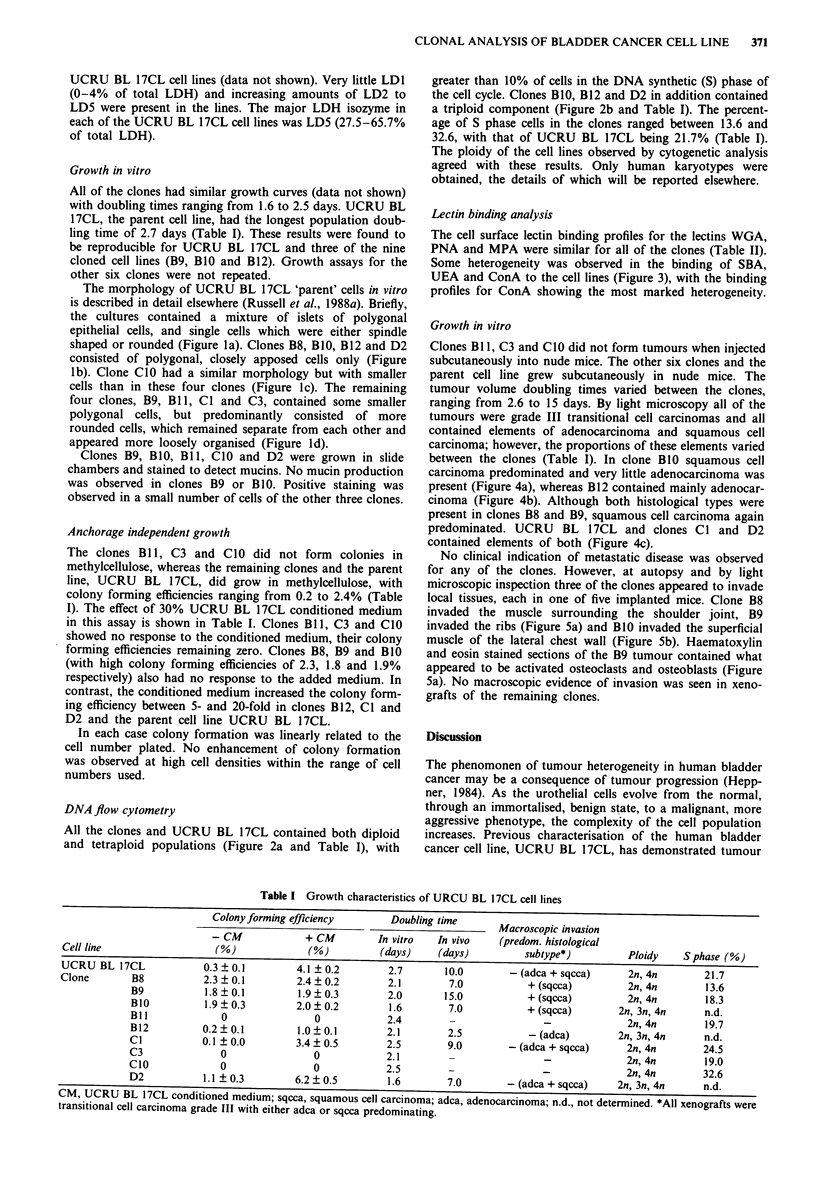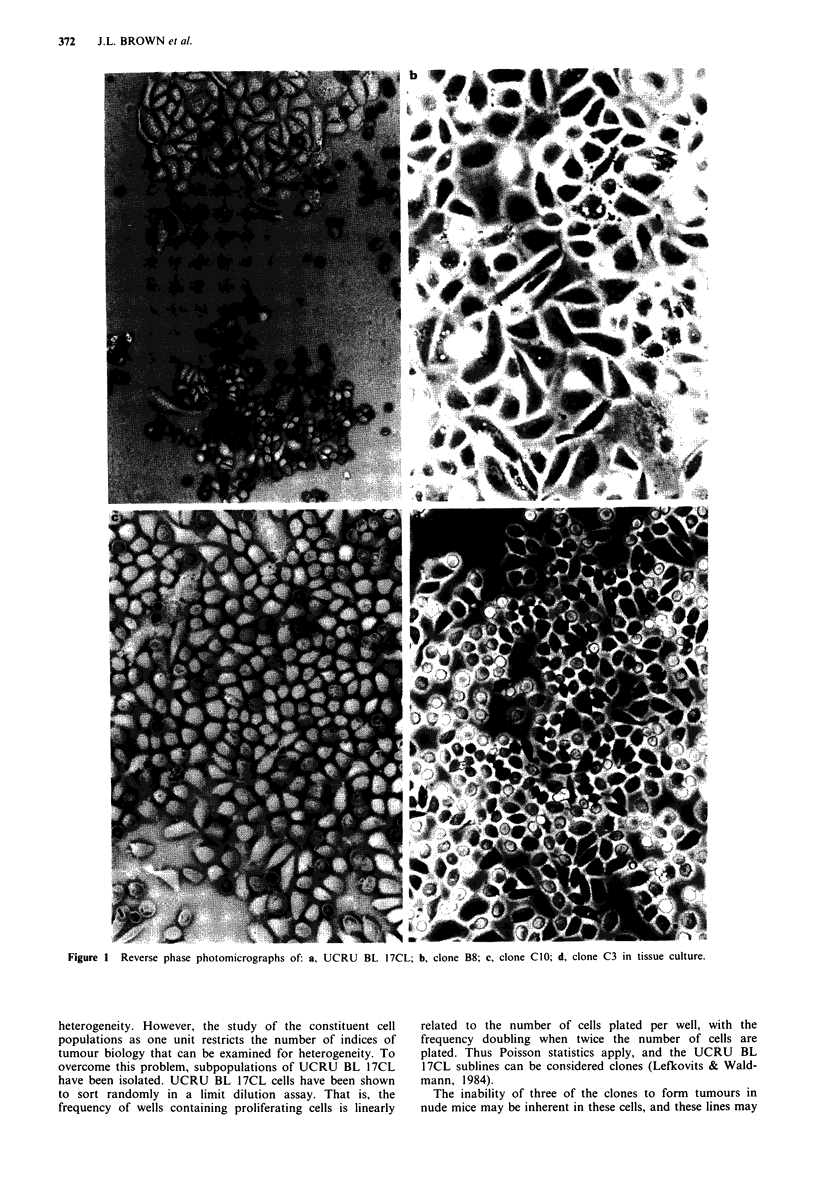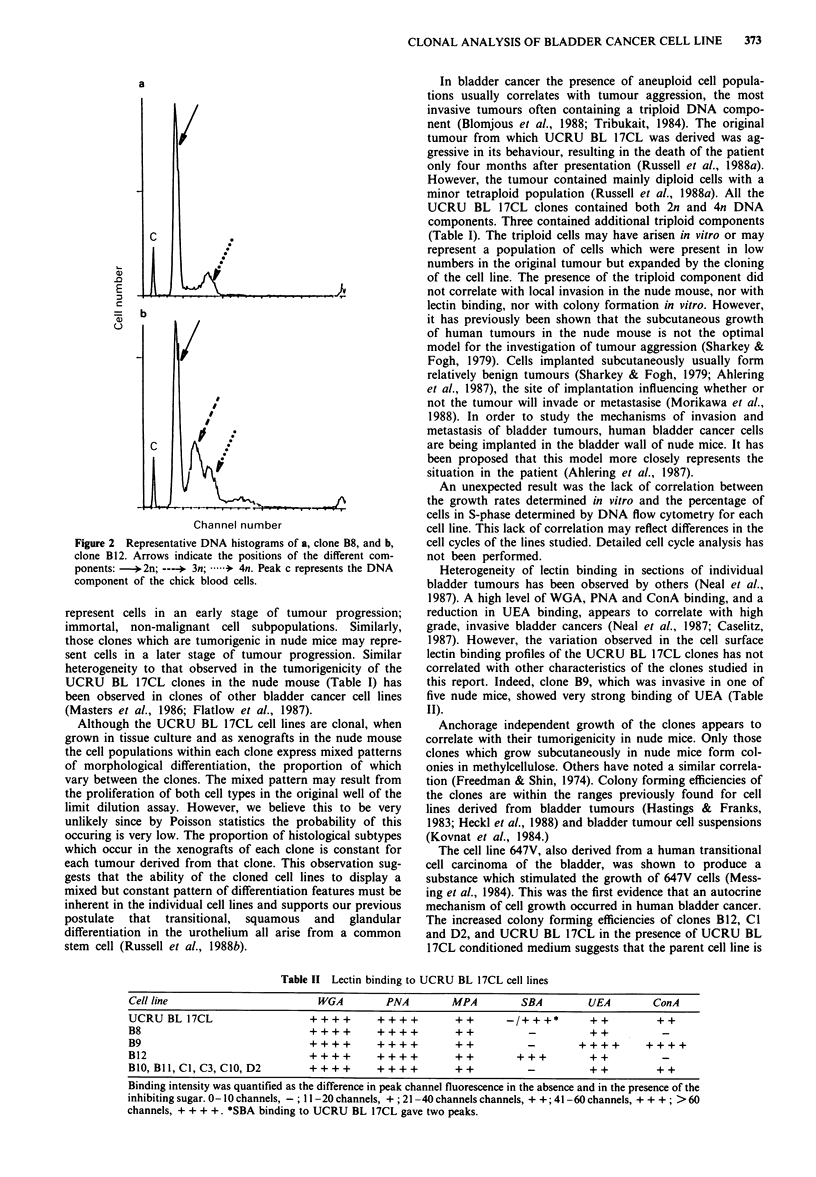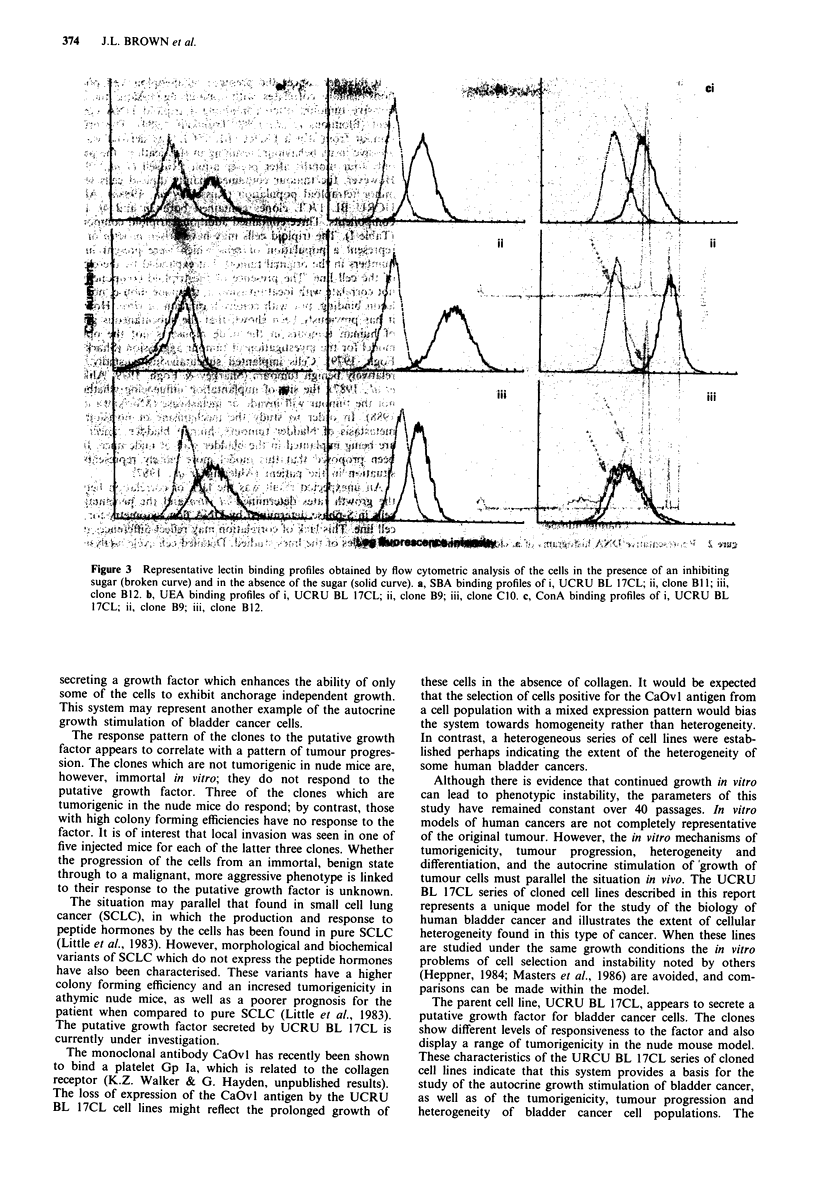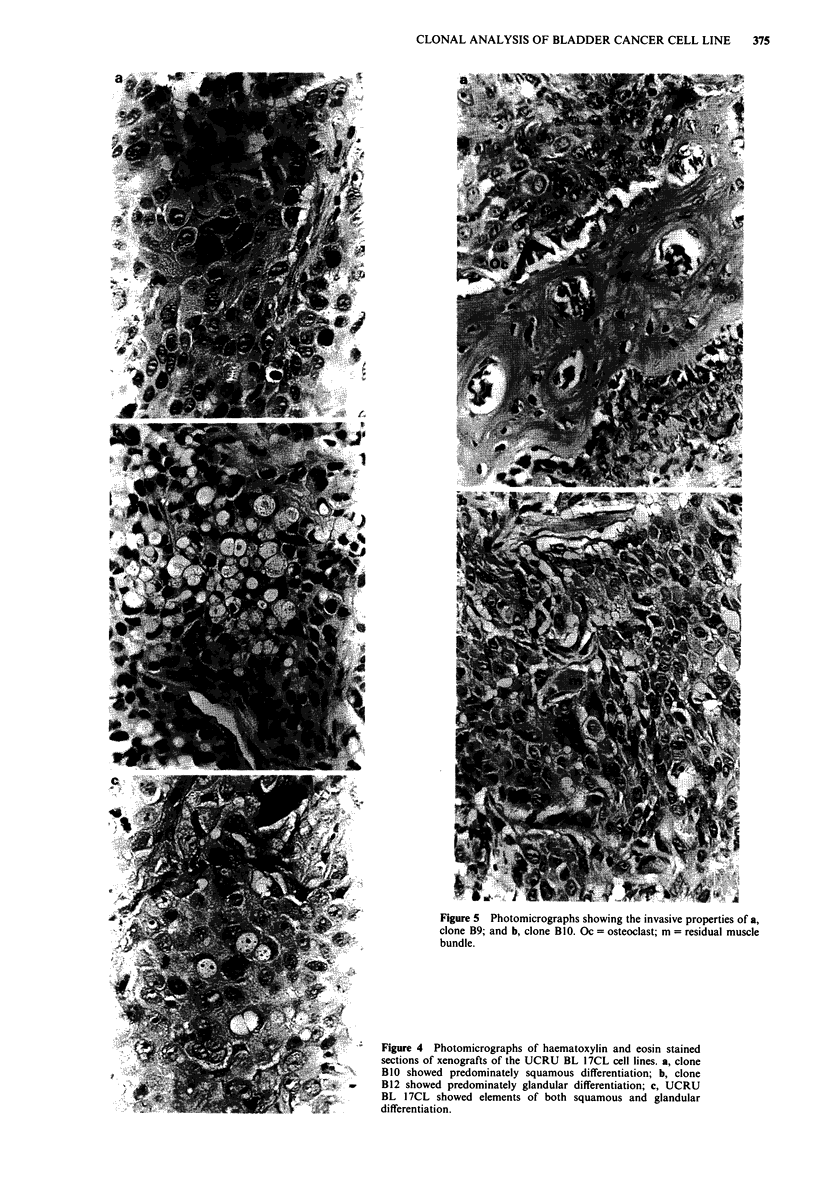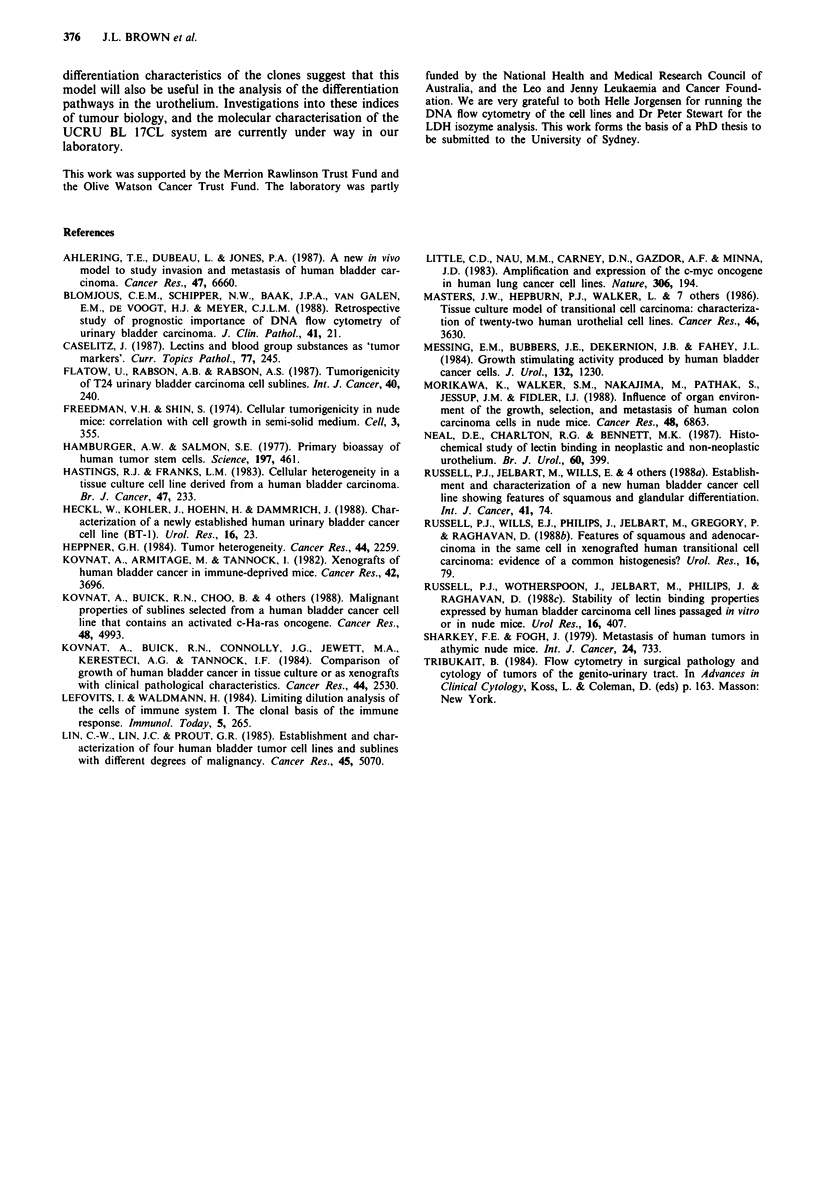# Clonal analysis of a bladder cancer cell line: an experimental model of tumour heterogeneity.

**DOI:** 10.1038/bjc.1990.81

**Published:** 1990-03

**Authors:** J. L. Brown, P. J. Russell, J. Philips, J. Wotherspoon, D. Raghavan

**Affiliations:** Urological Cancer Research Unit, Royal Prince Alfred Hospital, Camperdown, NSW, Australia.

## Abstract

**Images:**


Br. J. Cancer (1990), 61, 369-376 © Macmillan Press Ltd., 1990

### Clonal analysis of a bladder cancer cell line: an experimental model of tumour heterogeneity

J.L. Brown[1], P.J. Russell[1,*], J. Philips[2], J. Wotherspoon[3] & D. Raghavan[1]

[1]*Urological Cancer Research Unit, Royal Prince Alfred Hospital, Camperdown, 2050, and Department of Surgery, University of Sydney, 2006, NSW, Australia;* [2]*Department of Anatomical Pathology, Royal North Shore Hospital, St Leonards, 2065, NSW, Australia; and* [3]*Clinical Immunology Research Centre, University of Sydney, 2006, NSW, Australia.*

**Summary** The continuous cell line UCRU BL 17CL was derived from a human invasive bladder cancer and expresses elements of transitional, squamous and glandular differentiation. Nine clones of this line were established by limit dilution and have been extensively characterised. Only six of these clones grew subcutaneously in nude mice. Of these, three have exhibited local invasion, each in one of five implanted mice. Although all xenografts expressed transitional, squamous and glandular elements, different histological subtypes predominated within each clone. Only clones which grew in nude mice formed colonies in semi-solid medium, and each responded differently to the influence of medium that had been conditioned by the growth of UCRU BL 17CL, suggesting the possible secretion of a growth factor by these cells. The DNA content and lectin binding profiles of the clones also reflected the heterogeneity of the line. UCRU BL 17CL and the nine clones provide a unique model for the study of tumour heterogeneity, progression and differentiation, and the potential autocrine regulation of growth of bladder cancer.

In human bladder cancer, and in many other cancer types, the cell population within a single neoplasm is often heterogeneous. Tumours which appear homogeneous by light microscopy may contain cells with very different abilities to invade or metastasise or with varying degrees of sensitivity to cytotoxic agents and irradiation. Tumour progression (the evolution of the cells from a normal through to a malignant, more aggressive state) and the development of genetic instability are two factors which may give rise to tumour heterogeneity (Heppner, 1984). The establishment of *in vitro* cell lines derived from human cancers provides a model for the study of tumour progression and heterogeneity.

More than 30 cell lines have been established from human transitional cell carcinoma of the bladder (Lin *et al.*, 1985). Although many of these lines have been extensively studied, there have been few reports of the isolation and characterisation of individual cell subpopulations within them (Hastings & Franks, 1983; Lin *et al.*, 1985; Masters *et al.*, 1986; Kovnat *et al.*, 1988).

The continuous cell line UCRU BL 17CL was established from a human invasive (stage T4b) transitional cell carcinoma of the bladder (Russell *et al.*, 1988*a*). The line, when grown either *in vitro* or as xenografts in nude mice, expresses features of transitional, squamous and glandular differentiation (Russell *et al.*, 1988*a*, *b*). The histological and functional heterogeneity of this line *in vitro* has led to attempts to develop a series of cloned sublines of UCRU BL 17CL in order to determine whether the heterogeneity is due to the presence of different subpopulations or is inherent in the nature of the stem cells.

This report describes the development and extensive characterisation of nine clones of UCRU BL 17CL. We propose that this model illustrates the phenomonen of tumour heterogeneity, and provides a useful system for the study of tumorigenicity, tumour differentiation and progression, and sensitivity to treatment.

### Materials and methods

### *Cell line*

The line UCRU BL 17 was derived from a grade III, stage T4b transitional cell carcinoma of the bladder. The tumour was resistant to cisplatin and radiotherapy, and the patient died 4 months after presentation. The continuous cell line, UCRU BL 17CL, was derived from a xenograft established by implantation of a biopsy specimen, taken before treatment, into nude mice. The line produces mucin *in vitro*, and contains some cells expressing features of both squamous and glandular differentiation within the same cell (Russell *et al.*, 1988*a*, *b*).

### *Cell cultures*

All cell lines were maintained as previously described (Russell *et al.*, 1988*a*), but in a less complex culture medium: RPMI 1640 (Flow Laboratories, Australia), supplemented with 0.21% sodium bicarbonate, 4mM L-glutamine (Flow Laboratories) and 10% fetal calf serum (CSL, Australia and Cytosystems, Australia) heated to 56°C for 30 min.

### *Establishment of cloned sublines of UCRU BL 17CL*

For limit dilution adherent UCRU BL 17CL were harvested with 1mM EDTA (Flow Laboratories) at 37°C for 1-2 h. The cells were incubated on ice for 60 min with the monoclonal antibody, CaOv1 (kindly provided by G. Hayden, Clinical Immunology Research Centre, University of Sydney), raised against a panel of ovarian carcinoma cell lines and known to be reactive against UCRU BL 17CL cells (G. Hayden & K.Z. Walker, personal communication). Fluorescein conjugated sheep anti-mouse immunoglobulin (affinity purified FITC-SαMIg, diluted 1/50, Silenus, Australia) was used as a second antibody. This enabled the cells to be sorted on a fluorescence activated cell sorter (FACS). Green fluorescence was assessed by flow cytometric analysis on a FACS 440 (Becton Dickinson, Sunnyvale, CA, USA) using the 488 nm line of an argon ion laser (200 mW). Positive cells (two cells per well) were sorted into the wells of a 96-well plate (Flow Laboratories) filled with 0.25 ml of culture medium per well. Due to methodological problems, negative cells could not be sorted at this time. The number of cells per well was assessed under a phase contrast microscope and no wells were found to contain greater than two cells. Incubation was continued until cell proliferation was observed (approximately 2 weeks). Fourteen wells exhibiting cell growth were detected. From the frequency of positive wells and assuming random sorting, by the Poisson distribution these wells can be considered to contain clones (Lefkovits & Waldmann, 1984). Nine cell lines were established from nine of the 14 wells.

*Current address: Kanematsu Laboratories, Royal Prince Alfred Hospital, Camperdown, 2050, NSW, Australia.
Correspondence: J.L. Brown.
Received 4 April 1989; and in revised form 13 October 1989.

*Isozyme analysis*

Cultured cells were harvested with 0.25% trypsin, and suspended in 20 mM Tris.HCl pH7.5, 1% Triton X-100 (100 µl per $10^7$ cells). The suspension was incubated on ice for 20 min. After centrifugation at 1,000 *g* the supernatent was taken and stored at − 70°C until analysed. Lactate dehydrogenase (LDH) isozymes were analysed using electrophoresis by Dr Peter Stewart, Department of Biochemistry, Royal Prince Alfred Hospital, Sydney.

*Growth assay in vitro*

For each cell line, thirty 25 $cm^2$ tissue culture flasks (Corning, Australia) were seeded with $5 \times 10^4$ cells per flask, and incubated in 5% $O_2$. Cells from duplicate flasks were harvested with 0.25% trypsin (Cytosystems) and counted every second or third day until all flasks had been used. For each cell line at least five points were obtained while the cells were in an exponential phase of growth. Population doubling times were estimated after linear regression analysis of the data obtained.

*Anchorage independent growth*

Anchorage independent growth of cell lines was examined by the method of Hamburger and Salmon (1977). Briefly, underlayers of 0.5% agar in culture medium were established in six-well plates (35 $mm^2$ wells) (Flow Laboratories). Serial dilutions of trypsin harvested cells were suspended in 0.8% methylcellulose (Sigma, St Louis, MO, USA) in culture medium, and plating layers of 1 ml were poured over the preset agar underlayers in duplicate. The cultures were incubated in 5% $O_2$ for 14 days. Colonies containing more than 20 cells were counted under a phase contrast microscope and the colony forming efficiency of each cell line was calculated as the mean of the percentage of colonies formed per number of cells plated for each well.

*UCRU BL 17CL conditioned medium and colony forming efficiency*

The effect of UCRU BL 17CL conditioned medium on the colony forming efficiency of the cell lines was tested. The medium from a 70–80% confluent culture of UCRU BL 17CL was collected and non-adherent cells removed by centrifugation. The supernatant was filtered through a 0.2 µm filter unit (Millipore Products Division, Bedford, MA, USA) to ensure the removal of any residual tumour cells. The conditioned medium was used on the same day as prepared and was included in both underlayers and plating layers at a concentration of 30% v/v. Colony forming assays with and without UCRU BL 17CL conditioned medium for each clone were studied in parallel to allow the direct comparison of results.

*DNA flow cytometry*

Cells from tissue culture were stained in a solution of 1% v/v Triton X-100 (Packard, Downers Grove, IL, USA) containing 50 µg $ml^{-1}$ propidium iodide (Calbiochem, San Diego, CA, USA) and 800 µg $ml^{-1}$ Ribonuclease A (Sigma), and their DNA content was analysed using a FACS 440 (Becton Dickinson) with an argon ion laser excitation source at 488 nm (400 mW). A 620 nm long pass filter was used to collect the propidium iodide fluorescence. Chick blood cells were included in each sample as an internal standard to exclude staining and instrumental variability. The chick blood cell DNA peak was set at channel 15, and the data obtained analysed using a Consort 40 (Becton Dickinson).

*Lectin binding analysis*

Tissue culture cells were harvested by EDTA treatment and incubated with fluoresceinated lectins, and green fluorescence was assesed on a FACS 440 (Becton Dickinson) as previously described (Russell *et al.*, 1988*c*). Negative controls were assessed following binding of the lectin in the presence of the appropriate inhibiting sugar at a final concentration of 0.62 mM for *N*, *N*′, *N*″-triacetylchitotriose and 0.2 M for the remaining sugars. The lectins studied and the inhibitor for each were Jack Bean meal (*Conconavalin A*) (Con A) with mannose, Osage Orange (*Maclura pomifera*) (MPA) with D-galactose, peanut agglutinin (*Arachis hypogaea*) (PNA) with D-galactose, soy bean agglutinin (*Glycine max*) (SBA) with D-galactose, Gorse agglutinin (*Ulex europaeus*) (UEA) with fucose, and wheatgerm agglutinin (*Triticum vulgaris*) (WGA) with *N*,*N*′,*N*″-triacetylchitotriose. All lectins were obtained from E-Y Laboratories (San Mateo, CA, USA). Binding intensity was quantified as the difference in peak channel fluoresence (measured on a log scale) in the absence and in the presence of the inhibiting sugar.

*Growth in vivo*

Male BALB/c nu/nu ('nude') mice (Australian Atomic Energy Commission, Lucas Heights, NSW, Australia) were used to study the tumorigenicity of the cell lines. Their husbandry and maintenance have been reported previously (Russell *et al.*, 1988*b*). Tissue culture cells were injected subcutaneously over the scapular region of the nude mouse. For each line five mice were injected and each mouse received $5 \times 10^6$ cells in 0.1 ml phosphate buffered saline. Tumour growth was monitored twice weekly for periods up to 8 months. Opposite diamters ($D_1$ and $D_2$) were measured with calipers and the tumour volume (V) calculated by the formula (Kovnat *et al.*, 1982):

$$V = \pi/6\ (D_1 .\ D_2)^{3/2}$$

*Light microscopy*

Fragments of xenografted tumours were fixed in 10% buffered formalin, embedded in paraffin, sectioned and stained with haematoxylin and eosin, and with periodic acid Schiff plus diastase, mucicarmine and alcian blue for mucins. Tissue culture cells were grown to 70% confluence in slide chambers (LabTek Division, Miles Laboratories Inc., Naperville, IL, USA), fixed in 95% ethanol and tested for the presence of mucins with the stains listed above.

### Results

*Establishment of cloned cell lines*

Approximately 60% of UCRU BL 17CL cells (passage 15) were positive for the CaOv1 antigen (data not shown). Each well of a 96-well plate was seeded with two cells positive for the antigen using a fluorescence activated cell sorter. After two weeks, 14 wells were found to contain proliferating cells. The cells in one well stopped proliferating before reaching confluence. A further four wells were lost by bacterial contamination. The clones from the remaining nine wells were expanded into cell lines. Each cell line has been through at least 40 passages.

The nine clones, named according to the position of the well from which they were derived (vertical letter and horizontal number), and the parent cell line, UCRU BL 17CL, were later tested (10–15 passages after the limit dilution assay) for the presence of CaOv1 antigen expression so that the negative cells could be sorted. None of the cell lines expressed the antigen at this later stage, suggesting that the antigen is transiently expressed in prolonged culture.

*Isozyme analysis*

All of the cell lines tested contained only human LDH isozymes. The pattern of isozymes was similar in all of the

UCRU BL 17CL cell lines (data not shown). Very little LD1 (0–4% of total LDH) and increasing amounts of LD2 to LD5 were present in the lines. The major LDH isozyme in each of the UCRU BL 17CL cell lines was LD5 (27.5–65.7% of total LDH).

### *Growth in vitro*

All of the clones had similar growth curves (data not shown) with doubling times ranging from 1.6 to 2.5 days. UCRU BL 17CL, the parent cell line, had the longest population doubling time of 2.7 days (Table I). These results were found to be reproducible for UCRU BL 17CL and three of the nine cloned cell lines (B9, B10 and B12). Growth assays for the other six clones were not repeated.

The morphology of UCRU BL 17CL 'parent' cells *in vitro* is described in detail elsewhere (Russell *et al.*, 1988*a*). Briefly, the cultures contained a mixture of islets of polygonal epithelial cells, and single cells which were either spindle shaped or rounded (Figure 1a). Clones B8, B10, B12 and D2 consisted of polygonal, closely apposed cells only (Figure 1b). Clone C10 had a similar morphology but with smaller cells than in these four clones (Figure 1c). The remaining four clones, B9, B11, C1 and C3, contained some smaller polygonal cells, but predominantly consisted of more rounded cells, which remained separate from each other and appeared more loosely organised (Figure 1d).

Clones B9, B10, B11, C10 and D2 were grown in slide chambers and stained to detect mucins. No mucin production was observed in clones B9 or B10. Positive staining was observed in a small number of cells of the other three clones.

### *Anchorage independent growth*

The clones B11, C3 and C10 did not form colonies in methylcellulose, whereas the remaining clones and the parent line, UCRU BL 17CL, did grow in methylcellulose, with colony forming efficiencies ranging from 0.2 to 2.4% (Table I). The effect of 30% UCRU BL 17CL conditioned medium in this assay is shown in Table I. Clones B11, C3 and C10 showed no response to the conditioned medium, their colony forming efficiencies remaining zero. Clones B8, B9 and B10 (with high colony forming efficiencies of 2.3, 1.8 and 1.9% respectively) also had no response to the added medium. In contrast, the conditioned medium increased the colony forming efficiency between 5- and 20-fold in clones B12, C1 and D2 and the parent cell line UCRU BL 17CL.

In each case colony formation was linearly related to the cell number plated. No enhancement of colony formation was observed at high cell densities within the range of cell numbers used.

### *DNA flow cytometry*

All the clones and UCRU BL 17CL contained both diploid and tetraploid populations (Figure 2a and Table I), with greater than 10% of cells in the DNA synthetic (S) phase of the cell cycle. Clones B10, B12 and D2 in addition contained a triploid component (Figure 2b and Table I). The percentage of S phase cells in the clones ranged between 13.6 and 32.6, with that of UCRU BL 17CL being 21.7% (Table I). The ploidy of the cell lines observed by cytogenetic analysis agreed with these results. Only human karyotypes were obtained, the details of which will be reported elsewhere.

### *Lectin binding analysis*

The cell surface lectin binding profiles for the lectins WGA, PNA and MPA were similar for all of the clones (Table II). Some heterogeneity was observed in the binding of SBA, UEA and ConA to the cell lines (Figure 3), with the binding profiles for ConA showing the most marked heterogeneity.

### *Growth in vitro*

Clones B11, C3 and C10 did not form tumours when injected subcutaneously into nude mice. The other six clones and the parent cell line grew subcutaneously in nude mice. The tumour volume doubling times varied between the clones, ranging from 2.6 to 15 days. By light microscopy all of the tumours were grade III transitional cell carcinomas and all contained elements of adenocarcinoma and squamous cell carcinoma; however, the proportions of these elements varied between the clones (Table I). In clone B10 squamous cell carcinoma predominated and very little adenocarcinoma was present (Figure 4a), whereas B12 contained mainly adenocarcinoma (Figure 4b). Although both histological types were present in clones B8 and B9, squamous cell carcinoma again predominated. UCRU BL 17CL and clones C1 and D2 contained elements of both (Figure 4c).

No clinical indication of metastatic disease was observed for any of the clones. However, at autopsy and by light microscopic inspection three of the clones appeared to invade local tissues, each in one of five implanted mice. Clone B8 invaded the muscle surrounding the shoulder joint, B9 invaded the ribs (Figure 5a) and B10 invaded the superficial muscle of the lateral chest wall (Figure 5b). Haematoxylin and eosin stained sections of the B9 tumour contained what appeared to be activated osteoclasts and osteoblasts (Figure 5a). No macroscopic evidence of invasion was seen in xenografts of the remaining clones.

### Discussion

The phenomonen of tumour heterogeneity in human bladder cancer may be a consequence of tumour progression (Heppner, 1984). As the urothelial cells evolve from the normal, through an immortalised, benign state, to a malignant, more aggressive phenotype, the complexity of the cell population increases. Previous characterisation of the human bladder cancer cell line, UCRU BL 17CL, has demonstrated tumour

**Table I** Growth characteristics of URCU BL 17CL cell lines

| | | *Colony forming efficiency* | | *Doubling time* | | | | |
|---|---|---|---|---|---|---|---|---|
| *Cell line* | | *– CM (%)* | *+ CM (%)* | *In vitro (days)* | *In vivo (days)* | *Macroscopic invasion (predom. histological subtype*)* | *Ploidy* | *S phase (%)* |
| UCRU BL 17CL | | 0.3 ± 0.1 | 4.1 ± 0.2 | 2.7 | 10.0 | – (adca + sqcca) | 2*n*, 4*n* | 21.7 |
| Clone | B8 | 2.3 ± 0.1 | 2.4 ± 0.2 | 2.1 | 7.0 | + (sqcca) | 2*n*, 4*n* | 13.6 |
| | B9 | 1.8 ± 0.1 | 1.9 ± 0.3 | 2.0 | 15.0 | + (sqcca) | 2*n*, 4*n* | 18.3 |
| | B10 | 1.9 ± 0.3 | 2.0 ± 0.2 | 1.6 | 7.0 | + (sqcca) | 2*n*, 3*n*, 4*n* | n.d. |
| | B11 | 0 | 0 | 2.4 | – | – | 2*n*, 4*n* | 19.7 |
| | B12 | 0.2 ± 0.1 | 1.0 ± 0.1 | 2.1 | 2.5 | – (adca) | 2*n*, 3*n*, 4*n* | n.d. |
| | C1 | 0.1 ± 0.0 | 3.4 ± 0.5 | 2.5 | 9.0 | – (adca + sqcca) | 2*n*, 4*n* | 24.5 |
| | C3 | 0 | 0 | 2.1 | – | – | 2*n*, 4*n* | 19.0 |
| | C10 | 0 | 0 | 2.5 | – | – | 2*n*, 4*n* | 32.6 |
| | D2 | 1.1 ± 0.3 | 6.2 ± 0.5 | 1.6 | 7.0 | – (adca + sqcca) | 2*n*, 3*n*, 4*n* | n.d. |

CM, UCRU BL 17CL conditioned medium; sqcca, squamous cell carcinoma; adca, adenocarcinoma; n.d., not determined. *All xenografts were transitional cell carcinoma grade III with either adca or sqcca predominating.

**Figure 1** Reverse phase photomicrographs of: **a**, UCRU BL 17CL; **b**, clone B8; **c**, clone C10; **d**, clone C3 in tissue culture.

heterogeneity. However, the study of the constituent cell populations as one unit restricts the number of indices of tumour biology that can be examined for heterogeneity. To overcome this problem, subpopulations of UCRU BL 17CL have been isolated. UCRU BL 17CL cells have been shown to sort randomly in a limit dilution assay. That is, the frequency of wells containing proliferating cells is linearly related to the number of cells plated per well, with the frequency doubling when twice the number of cells are plated. Thus Poisson statistics apply, and the UCRU BL 17CL sublines can be considered clones (Lefkovits & Waldmann, 1984).

The inability of three of the clones to form tumours in nude mice may be inherent in these cells, and these lines may

**Figure 2** Representative DNA histograms of **a**, clone B8, and **b**, clone B12. Arrows indicate the positions of the different components: ——> 2n; ---> 3n; ·····> 4n. Peak c represents the DNA component of the chick blood cells.

represent cells in an early stage of tumour progression; immortal, non-malignant cell subpopulations. Similarly, those clones which are tumorigenic in nude mice may represent cells in a later stage of tumour progression. Similar heterogeneity to that observed in the tumorigenicity of the UCRU BL 17CL clones in the nude mouse (Table I) has been observed in clones of other bladder cancer cell lines (Masters *et al.*, 1986; Flatlow *et al.*, 1987).

Although the UCRU BL 17CL cell lines are clonal, when grown in tissue culture and as xenografts in the nude mouse the cell populations within each clone express mixed patterns of morphological differentiation, the proportion of which vary between the clones. The mixed pattern may result from the proliferation of both cell types in the original well of the limit dilution assay. However, we believe this to be very unlikely since by Poisson statistics the probability of this occuring is very low. The proportion of histological subtypes which occur in the xenografts of each clone is constant for each tumour derived from that clone. This observation suggests that the ability of the cloned cell lines to display a mixed but constant pattern of differentiation features must be inherent in the individual cell lines and supports our previous postulate that transitional, squamous and glandular differentiation in the urothelium all arise from a common stem cell (Russell *et al.*, 1988*b*).

In bladder cancer the presence of aneuploid cell populations usually correlates with tumour aggression, the most invasive tumours often containing a triploid DNA component (Blomjous *et al.*, 1988; Tribukait, 1984). The original tumour from which UCRU BL 17CL was derived was aggressive in its behaviour, resulting in the death of the patient only four months after presentation (Russell *et al.*, 1988*a*). However, the tumour contained mainly diploid cells with a minor tetraploid population (Russell *et al.*, 1988*a*). All the UCRU BL 17CL clones contained both 2*n* and 4*n* DNA components. Three contained additional triploid components (Table I). The triploid cells may have arisen *in vitro* or may represent a population of cells which were present in low numbers in the original tumour but expanded by the cloning of the cell line. The presence of the triploid component did not correlate with local invasion in the nude mouse, nor with lectin binding, nor with colony formation *in vitro*. However, it has previously been shown that the subcutaneous growth of human tumours in the nude mouse is not the optimal model for the investigation of tumour aggression (Sharkey & Fogh, 1979). Cells implanted subcutaneously usually form relatively benign tumours (Sharkey & Fogh, 1979; Ahlering *et al.*, 1987), the site of implantation influencing whether or not the tumour will invade or metastasise (Morikawa *et al.*, 1988). In order to study the mechanisms of invasion and metastasis of bladder tumours, human bladder cancer cells are being implanted in the bladder wall of nude mice. It has been proposed that this model more closely represents the situation in the patient (Ahlering *et al.*, 1987).

An unexpected result was the lack of correlation between the growth rates determined *in vitro* and the percentage of cells in S-phase determined by DNA flow cytometry for each cell line. This lack of correlation may reflect differences in the cell cycles of the lines studied. Detailed cell cycle analysis has not been performed.

Heterogeneity of lectin binding in sections of individual bladder tumours has been observed by others (Neal *et al.*, 1987). A high level of WGA, PNA and ConA binding, and a reduction in UEA binding, appears to correlate with high grade, invasive bladder cancers (Neal *et al.*, 1987; Caselitz, 1987). However, the variation observed in the cell surface lectin binding profiles of the UCRU BL 17CL clones has not correlated with other characteristics of the clones studied in this report. Indeed, clone B9, which was invasive in one of five nude mice, showed very strong binding of UEA (Table II).

Anchorage independent growth of the clones appears to correlate with their tumorigenicity in nude mice. Only those clones which grow subcutaneously in nude mice form colonies in methylcellulose. Others have noted a similar correlation (Freedman & Shin, 1974). Colony forming efficiencies of the clones are within the ranges previously found for cell lines derived from bladder tumours (Hastings & Franks, 1983; Heckl *et al.*, 1988) and bladder tumour cell suspensions (Kovnat *et al.*, 1984.)

The cell line 647V, also derived from a human transitional cell carcinoma of the bladder, was shown to produce a substance which stimulated the growth of 647V cells (Messing *et al.*, 1984). This was the first evidence that an autocrine mechanism of cell growth occurred in human bladder cancer. The increased colony forming efficiencies of clones B12, C1 and D2, and UCRU BL 17CL in the presence of UCRU BL 17CL conditioned medium suggests that the parent cell line is

**Table II** Lectin binding to UCRU BL 17CL cell lines

| *Cell line* | *WGA* | *PNA* | *MPA* | *SBA* | *UEA* | *ConA* |
|---|---|---|---|---|---|---|
| UCRU BL 17CL | + + + + | + + + + | + + | – / + + + * | + + | + + |
| B8 | + + + + | + + + + | + + | – | + + | – |
| B9 | + + + + | + + + + | + + | – | + + + + | + + + + |
| B12 | + + + + | + + + + | + + | + + + | + + | – |
| B10, B11, C1, C3, C10, D2 | + + + + | + + + + | + + | – | + + | + + |

Binding intensity was quantified as the difference in peak channel fluorescence in the absence and in the presence of the inhibiting sugar. 0–10 channels, – ; 11–20 channels, + ; 21–40 channels channels, + + ; 41–60 channels, + + + ; >60 channels, + + + +. *SBA binding to UCRU BL 17CL gave two peaks.

**Figure 3** Representative lectin binding profiles obtained by flow cytometric analysis of the cells in the presence of an inhibiting sugar (broken curve) and in the absence of the sugar (solid curve). **a**, SBA binding profiles of i, UCRU BL 17CL; ii, clone B11; iii, clone B12. **b**, UEA binding profiles of i, UCRU BL 17CL; ii, clone B9; iii, clone C10. **c**, ConA binding profiles of i, UCRU BL 17CL; ii, clone B9; iii, clone B12.

secreting a growth factor which enhances the ability of only some of the cells to exhibit anchorage independent growth. This system may represent another example of the autocrine growth stimulation of bladder cancer cells.

The response pattern of the clones to the putative growth factor appears to correlate with a pattern of tumour progression. The clones which are not tumorigenic in nude mice are, however, immortal *in vitro*; they do not respond to the putative growth factor. Three of the clones which are tumorigenic in the nude mice do respond; by contrast, those with high colony forming efficiencies have no response to the factor. It is of interest that local invasion was seen in one of five injected mice for each of the latter three clones. Whether the progression of the cells from an immortal, benign state through to a malignant, more aggressive phenotype is linked to their response to the putative growth factor is unknown.

The situation may parallel that found in small cell lung cancer (SCLC), in which the production and response to peptide hormones by the cells has been found in pure SCLC (Little *et al.*, 1983). However, morphological and biochemical variants of SCLC which do not express the peptide hormones have also been characterised. These variants have a higher colony forming efficiency and an incresed tumorigenicity in athymic nude mice, as well as a poorer prognosis for the patient when compared to pure SCLC (Little *et al.*, 1983). The putative growth factor secreted by UCRU BL 17CL is currently under investigation.

The monoclonal antibody CaOv1 has recently been shown to bind a platelet Gp Ia, which is related to the collagen receptor (K.Z. Walker & G. Hayden, unpublished results). The loss of expression of the CaOv1 antigen by the UCRU BL 17CL cell lines might reflect the prolonged growth of these cells in the absence of collagen. It would be expected that the selection of cells positive for the CaOv1 antigen from a cell population with a mixed expression pattern would bias the system towards homogeneity rather than heterogeneity. In contrast, a heterogeneous series of cell lines were established perhaps indicating the extent of the heterogeneity of some human bladder cancers.

Although there is evidence that continued growth *in vitro* can lead to phenotypic instability, the parameters of this study have remained constant over 40 passages. *In vitro* models of human cancers are not completely representative of the original tumour. However, the *in vitro* mechanisms of tumorigenicity, tumour progression, heterogeneity and differentiation, and the autocrine stimulation of 'growth of tumour cells must parallel the situation *in vivo*. The UCRU BL 17CL series of cloned cell lines described in this report represents a unique model for the study of the biology of human bladder cancer and illustrates the extent of cellular heterogeneity found in this type of cancer. When these lines are studied under the same growth conditions the *in vitro* problems of cell selection and instability noted by others (Heppner, 1984; Masters *et al.*, 1986) are avoided, and comparisons can be made within the model.

The parent cell line, UCRU BL 17CL, appears to secrete a putative growth factor for bladder cancer cells. The clones show different levels of responsiveness to the factor and also display a range of tumorigenicity in the nude mouse model. These characteristics of the URCU BL 17CL series of cloned cell lines indicate that this system provides a basis for the study of the autocrine growth stimulation of bladder cancer, as well as of the tumorigenicity, tumour progression and heterogeneity of bladder cancer cell populations. The

**Figure 5** Photomicrographs showing the invasive properties of **a**, clone B9; and **b**, clone B10. Oc = osteoclast; m = residual muscle bundle.

**Figure 4** Photomicrographs of haematoxylin and eosin stained sections of xenografts of the UCRU BL 17CL cell lines. **a**, clone B10 showed predominately squamous differentiation; **b**, clone B12 showed predominately glandular differentiation; **c**, UCRU BL 17CL showed elements of both squamous and glandular differentiation.

differentiation characteristics of the clones suggest that this model will also be useful in the analysis of the differentiation pathways in the urothelium. Investigations into these indices of tumour biology, and the molecular characterisation of the UCRU BL 17CL system are currently under way in our laboratory.

This work was supported by the Merrion Rawlinson Trust Fund and the Olive Watson Cancer Trust Fund. The laboratory was partly funded by the National Health and Medical Research Council of Australia, and the Leo and Jenny Leukaemia and Cancer Foundation. We are very grateful to both Helle Jorgensen for running the DNA flow cytometry of the cell lines and Dr Peter Stewart for the LDH isozyme analysis. This work forms the basis of a PhD thesis to be submitted to the University of Sydney.

### References

AHLERING, T.E., DUBEAU, L. & JONES, P.A. (1987). A new *in vivo* model to study invasion and metastasis of human bladder carcinoma. *Cancer Res.*, **47**, 6660.

BLOMJOUS, C.E.M., SCHIPPER, N.W., BAAK, J.P.A., VAN GALEN, E.M., DE VOOGT, H.J. & MEYER, C.J.L.M. (1988). Retrospective study of prognostic importance of DNA flow cytometry of urinary bladder carcinoma. *J. Clin. Pathol.*, **41**, 21.

CASELITZ, J. (1987). Lectins and blood group substances as 'tumor markers'. *Curr. Topics Pathol.*, **77**, 245.

FLATOW, U., RABSON, A.B. & RABSON, A.S. (1987). Tumorigenicity of T24 urinary bladder carcinoma cell sublines. *Int. J. Cancer*, **40**, 240.

FREEDMAN, V.H. & SHIN, S. (1974). Cellular tumorigenicity in nude mice: correlation with cell growth in semi-solid medium. *Cell*, **3**, 355.

HAMBURGER, A.W. & SALMON, S.E. (1977). Primary bioassay of human tumor stem cells. *Science*, **197**, 461.

HASTINGS, R.J. & FRANKS, L.M. (1983). Cellular heterogeneity in a tissue culture cell line derived from a human bladder carcinoma. *Br. J. Cancer*, **47**, 233.

HECKL, W., KOHLER, J., HOEHN, H. & DAMMRICH, J. (1988). Characterization of a newly established human urinary bladder cancer cell line (BT-1). *Urol. Res.*, **16**, 23.

HEPPNER, G.H. (1984). Tumor heterogeneity. *Cancer Res.*, **44**, 2259.

KOVNAT, A., ARMITAGE, M. & TANNOCK, I. (1982). Xenografts of human bladder cancer in immune-deprived mice. *Cancer Res.*, **42**, 3696.

KOVNAT, A., BUICK, R.N., CHOO, B. & 4 others (1988). Malignant properties of sublines selected from a human bladder cancer cell line that contains an activated c-Ha-ras oncogene. *Cancer Res.*, **48**, 4993.

KOVNAT, A., BUICK, R.N., CONNOLLY, J.G., JEWETT, M.A., KERESTECI, A.G. & TANNOCK, I.F. (1984). Comparison of growth of human bladder cancer in tissue culture or as xenografts with clinical pathological characteristics. *Cancer Res.*, **44**, 2530.

LEFOVITS, I. & WALDMANN, H. (1984). Limiting dilution analysis of the cells of immune system I. The clonal basis of the immune response. *Immunol. Today*, **5**, 265.

LIN, C.-W., LIN, J.C. & PROUT, G.R. (1985). Establishment and characterization of four human bladder tumor cell lines and sublines with different degrees of malignancy. *Cancer Res.*, **45**, 5070.

LITTLE, C.D., NAU, M.M., CARNEY, D.N., GAZDOR, A.F. & MINNA, J.D. (1983). Amplification and expression of the c-myc oncogene in human lung cancer cell lines. *Nature*, **306**, 194.

MASTERS, J.W., HEPBURN, P.J., WALKER, L. & 7 others (1986). Tissue culture model of transitional cell carcinoma: characterization of twenty-two human urothelial cell lines. *Cancer Res.*, **46**, 3630.

MESSING, E.M., BUBBERS, J.E., DEKERNION, J.B. & FAHEY, J.L. (1984). Growth stimulating activity produced by human bladder cancer cells. *J. Urol.*, **132**, 1230.

MORIKAWA, K., WALKER, S.M., NAKAJIMA, M., PATHAK, S., JESSUP, J.M. & FIDLER, I.J. (1988). Influence of organ environment of the growth, selection, and metastasis of human colon carcinoma cells in nude mice. *Cancer Res.*, **48**, 6863.

NEAL, D.E., CHARLTON, R.G. & BENNETT, M.K. (1987). Histochemical study of lectin binding in neoplastic and non-neoplastic urothelium. *Br. J. Urol.*, **60**, 399.

RUSSELL, P.J., JELBART, M., WILLS, E. & 4 others (1988*a*). Establishment and characterization of a new human bladder cancer cell line showing features of squamous and glandular differentiation. *Int. J. Cancer*, **41**, 74.

RUSSELL, P.J., WILLS, E.J., PHILIPS, J., JELBART, M., GREGORY, P. & RAGHAVAN, D. (1988*b*). Features of squamous and adenocarcinoma in the same cell in xenografted human transitional cell carcinoma: evidence of a common histogenesis? *Urol. Res.*, **16**, 79.

RUSSELL, P.J., WOTHERSPOON, J., JELBART, M., PHILIPS, J. & RAGHAVAN, D. (1988*c*). Stability of lectin binding properties expressed by human bladder carcinoma cell lines passaged *in vitro* or in nude mice. *Urol Res.*, **16**, 407.

SHARKEY, F.E. & FOGH, J. (1979). Metastasis of human tumors in athymic nude mice. *Int. J. Cancer*, **24**, 733.

TRIBUKAIT, B. (1984). Flow cytometry in surgical pathology and cytology of tumors of the genito-urinary tract. In *Advances in Clinical Cytology*, Koss, L. & Coleman, D. (eds) p. 163. Masson: New York.